# Towards Digital Twins of the Oceans: The Potential of Machine Learning for Monitoring the Impacts of Offshore Wind Farms on Marine Environments

**DOI:** 10.3390/s23104581

**Published:** 2023-05-09

**Authors:** Janina Schneider, André Klüner, Oliver Zielinski

**Affiliations:** 1 Research Department Marine Perception, German Research Center for Artificial Intelligence, Marie-Curie-Straße 1, 26129 Oldenburg, Germany; andre.kluener@dfki.de (A.K.); oliver.zielinski@io-warnemuende.de (O.Z.); 2 Institute for Chemistry and Biology of the Marine Environment, Carl von Ossietzky University of Oldenburg, Schleusenstraße 1, 26382 Wilhelmshaven, Germany; 3 Leibniz Institute for Baltic Sea Research Warnemünde, Seestraße 15, 18119 Rostock, Germany

**Keywords:** artificial intelligence, anomaly detection, missing value imputation, marine ecosystem, earth observation, North Sea, SDG, remote sensing, green energy

## Abstract

With an increasing number of offshore wind farms, monitoring and evaluating the effects of the wind turbines on the marine environment have become important tasks. Here we conducted a feasibility study with the focus on monitoring these effects by utilizing different machine learning methods. A multi-source dataset for a study site in the North Sea is created by combining satellite data, local in situ data and a hydrodynamic model. The machine learning algorithm DTWkNN, which is based on dynamic time warping and *k*-nearest neighbor, is used for multivariate time series data imputation. Subsequently, unsupervised anomaly detection is performed to identify possible inferences in the dynamic and interdepending marine environment around the offshore wind farm. The anomaly results are analyzed in terms of location, density and temporal variability, granting access to information and building a basis for explanation. Temporal detection of anomalies with COPOD is found to be a suitable method. Actionable insights are the direction and magnitude of potential effects of the wind farm on the marine environment, depending on the wind direction. This study works towards a digital twin of offshore wind farms and provides a set of methods based on machine learning to monitor and evaluate offshore wind farm effects, supporting stakeholders with information for decision making on future maritime energy infrastructures.

## 1. Introduction

Offshore wind farms (OWFs) represent one of the key components of the energy transition, which makes their sustainable use crucial for maintaining a healthy marine environment. All across Europe’s coastal waters and shelf seas, including the Exclusive Economic Zone of Germany, many wind farms have been constructed or are under planning, scaling up to over 5400 wind turbines [1]. Their safe operation and resilience are key to the European supply with green energy [2].

While energy monitoring and predictive modeling of wind energy harvesting are operationally established, there is a lack in understanding, monitoring and managing effects on the marine ecosystem in and around OWFs. Many studies focus on processes in the atmospheric layers, including air–sea interactions [3,4]. In particular, the influences of wind wakes are examined, which occur downstream of OWFs and exhibit a reduced wind speed, leading to possible upwelling/downwelling cells in the water [5,6]. Recent research showed that OWFs also strongly affect the stratification and the pelagic ecosystem [7,8]. The tidal currents in the North Sea pass the OWF foundations and are forced around them, resulting in enhanced vertical mixing as turbulences behind the structures [9]. By examination of empirical data from the North Sea, the local seasonal water column stratification in OWF areas is found to be decreased, leading to a change in the transport and distribution of nutrients [7]. These effects may also have an impact on a larger, basin-wide scale, especially when large-scale wind energy development scenarios are implemented [10]. Ideally, these effects of OWFs on the marine environment should be known and accounted for not only in the construction and running phase of OWFs but also in the planning phase of new wind farms, in order to minimize harmful impacts on the environment. Yet, no baseline is established. Most of the work is based on predictions from modeling analyses with a specific focus and some on empirical data, e.g., bio-physical data measured with a remotely operated vehicle [7]. The said study was conducted by towing it through non-operating OWFs and, additionally, recording a baseline before the wind farm was built. Such measurement campaigns are cost and time consuming and for OWFs often limited due to permission restrictions. Moreover, they represent a snap-shot instead of a permanent and sustainable technique. Data-driven techniques based on and supported by such studies can advance the development of these monitoring methods.

A potential tool for such applications is digital twins. A concept from product lifecycle management [11,12], it recently also attracted attention for environmental applications. Especially for tackling environmental and climate challenges, as committed to in the European Green Deal [2], it is discussed with following projects such as Destination Earth [13] and to support the UN Sustainable Development Goals (SDGs) [14]. As a digital representation of a real entity, e.g., the ocean, it makes the data and information accessible for everyone while being able to look into the past, present and future. In addition, testing scenarios enable questions to be asked by users and stakeholders. With consequent actions based on the digital results, the real twin can change and adapt. An important part for the development of digital twins for the natural environment will be the use of the potential of Artificial Intelligence (AI) [15]. It will among other things support the handling of multivariate data, processing steps and predictions.

Here, we present a feasibility study on the utilization of machine learning for multivariate data imputation and subsequent anomaly detection in marine Earth Observation data, combined with local data sources and models for monitoring OWF effects on the marine ecosystem. It is based on the anomalies in the data, which describe the deviation from normal behavior. For each step in the pipeline, appropriate methods are researched and finally one is implemented to generate a first working demonstrator. The focus regarding the study site is hereby on the North Sea, which will be one of the main locations of interest for the planned offshore wind energy expansion in Europe [16]. The main advantage of this approach is the possibility for automation and easy implementation, as it is based on public and available data, enabling continuous monitoring. This paper is organized as follows: Section 2 describes the study area around the OWF DanTysk and the available data, as well as the proposed workflow of the feasibility study. In Section 3, results of missing value imputation and anomaly detection are presented and discussed with a focus on explainability. Finally, a conclusion is given in Section 4.

## 2. Materials and Methods

### 2.1. Study Site and Data

**Figure 1:**
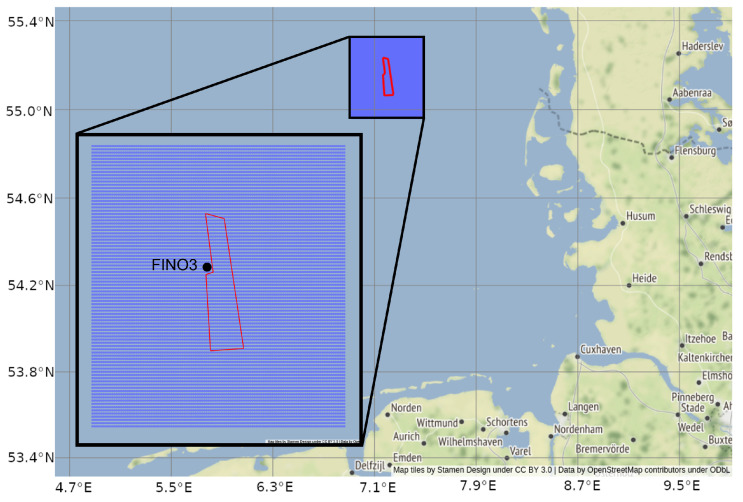
Location of the wind farm DanTysk (red) in the North Sea. The study site as data grid is visualized in purple. In situ measurements are available from the FINO3 station, directly at the wind farm (see inserted box for details).

This study is placed in the North Sea with the focus on the DanTysk wind farm located in the north of the German Bight, 70 km west of the island Sylt (Figure 1).

The study area was chosen due to its remote location with only two additional operating wind farms in the closer neighborhood (about 15 km and 30 km distance), so that possible influences through OWF cluster structures can be neglected. Furthermore, the research platform FINO3 (http://fino3.de accessed on 13 March 2023) is directly placed at DanTysk, providing freely available meteorological and oceanographic in situ data. The study site is located between 54.97–55.32 N and 6.92–7.47 E and covers an area of more than 1300 km² with 80 DanTysk wind turbines in the center extending over around 71 km². As a time frame, the month of May 2018 was selected for this feasability study considering its favorable meteorological properties with only six days for the North Sea with partly cloud coverage. Cloudless days are preferred, since clouds represent a major challenge in processing and therefore availability of satellite products.

Different data sources for meteorological, oceanographic and bio-optical data need to be considered for inclusion. A detailed overview of the data and their properties is shown in Table 1.

Earth Observation data from Sentinel-3A was used, downloading Level-2 Ocean Color products OL_2_WFR from the Ocean and Land Color Instrument (OLCI) in full resolution on a daily basis. From this Total Suspended Matter (TSM) and Chlorophyll (CHL), both based on neural network algorithms [17], were extracted. From the Sea and Land Surface Temperature Radiometer (SLSTR) the Sea Surface Temperature (SST) products were utilized.

Local data sources are restricted, due to the remoteness of an OWF. There are two FINO research platforms in the North Sea, which are operated by the FuE Center FH Kiel GmbH. Directly located at OWF sites, these research platforms collect meteorological and oceanographic data and therefore make it possible to research different aspects of OWFs. For this use case FINO3 was chosen as it is located at the DanTysk wind farm (N 55.195 E 7.158). Data were accessed via a database provided by the Federal Maritime and Hydrographic Agency (BSH). The parameters wind direction at 29 m height and wind speed at 31 m height with a temporal resolution of 10 min were downloaded.

For the North Sea the numerical model system HBMnoku operated by BSH is available. It includes a hydrodynamical model, a biogeochemical model and a data assimilation component [18]. The spatial resolution in the German Bight is 900 m horizontal with up to 25 layers vertically. The temporal resolution differs depending on the parameter. For this study, current velocity *u*, *v* and current direction at 0 m with a temporal resolution of 15 min, and water temperature $T_{0}$ and salinity $S_{0}$ at 0 m with hourly values were selected. At 0 m, these values describe the state of the sea surface.

### 2.2. Workflow

**Figure 2:**
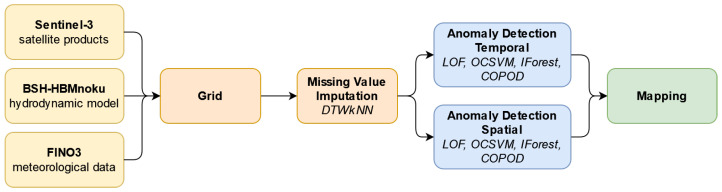
Architecture of the study from input data provided by different sources, over preprocessing steps to visualization of the anomaly detection results, whereby a temporal and a spatial anomaly detection method is tested. The AI algorithms used are highlighted for the missing value imputation and anomaly detection.

The workflow in this feasibility study follows an architecture from data acquisition, over processing steps and models, to visualization. The focus is hereby on the potential of machine learning methods for preprocessing and modeling (Figure 2).

Input data are gathered from different marine data sources including remote sensing, models and in situ measurements. To overcome spatial and temporal discrepancies, the data are fused. For this purpose, the data were stretched onto a common grid within the chosen study area with 0.004° as distance between the individual grid points. To fit the data with different spatial resolutions on the common grid, the value closest to each grid point is used, similar to a *k*-nearest neighbor (kNN) algorithm with *k* = 1 [19]. The distance in kilometers for two latitude–longitude pairs is here computed with the Haversine formula. For every grid point the system searches for the nearest data point and adopts the value. When the nearest neighbor is more than 1 km away, no value is passed to the grid point. Compared to the satellite and model data, the meteorological data from FINO3 just contains the information of a point measurement and therefore every grid point adopts the same values. Regarding the temporal span of the grid, the overflight time of the Sentinel-3A satellite was taken as metric for daily values. For the meteorological and model data an average value for this time window was calculated. Overall, the complete dataset consists of around 12,000 grid points each containing the daily multivariate data from the different sources.

Events or anomalies describe the deviation from normal behavior [20]. Their detection can give insight and help understand the underlying processes. Often these anomalies occur in different features simultaneously. For the anomaly detection algorithms, a complete dataset is needed. Therefore, missing single values and larger gaps in the data must be filled before passing it to the anomaly detector. This processing step is handled by the DTWkNN algorithm [21]. It is a kNN algorithm, which uses dynamic time warping (DTW) as a distance measure. With this algorithm it is possible to fill large gaps in time series data, as it relies on the similarity of comparing different time windows and therefore enabling it to match the temporal variability of time dependent data. It can be used as an ensemble method as well, by combining multiple single models with different hyperparameter settings. As it handles time dependent data, the time series for each individual grid point is created while reading the daily datasets for the whole month. On these small time series the missing values are imputed with the implemented algorithm.

Subsequently, the anomaly detection is conducted on the dataset generated as described previously. Two different approaches are tested: temporal and spatial. While temporal anomaly detection looks at the time series of each grid point individually and calculates the anomaly scores depending on the data for this particular grid point from different dates of the month, the spatial approach determines the anomalies by giving all grid points for a specific day as input to the detector, without the time component. Different unsupervised anomaly detectors from the Python toolkit PyOD [22] are evaluated: Local Outlier Factor (LOF), Isolation Forest (IForest), One-Class Support Vector Machine (OCSVM) and Copula-Based Outlier Detection (COPOD). LOF [23] is a proximity-based detector measuring the kNN-based local deviation of density to its neighbors. IForest [24] is an ensemble based on decision trees, where one feature and finally the split value based on the feature’s range are chosen randomly, in order to isolate the observation. OCSVM [25] is a linear model maximizing the margin between the origin and the data points. COPOD [26] is a probabilistic outlier detection algorithm based on empirical copula models. A more detailed description can be found in the respective publications, as well as comparisons in [27,28,29]. The resulting anomaly scores are an indicator to which degree the data point is an outlier compared to the remaining values.

Finally, the anomaly scores are visualized for each day on a map around the wind farm. Data clusters with high scores can then be identified as anomalous areas and therefore as possible effects of the OWF which need to be investigated further. For these results, the different approaches and in addition different combinations of input variables are compared. A suitable algorithm is selected by comparison of their behavior over the temporal course. The examination of the anomalies is performed under consideration of different aspects. First, the anomaly scores are ranked and only the highest values of a daily subset are utilized for further analysis. The amount is scaled with the standard deviation to avoid favoring anomalous over basic subsets. After specification through sorting by the highest values and normalization between 0 and 1, the temporal extent is evaluated by dividing the data into time windows with similar meteorological conditions based on wind direction and averaging the values. Then, a measure for size and spatial extent of the anomalies is generated by the calculation of the centroid C of the data with *n* remaining grid points ($\varphi_{n},\lambda_{n}$), assuming a plane surface, as

(1) $$\begin{array}{c} C=\left(\frac{\sum_{i=1}^{n}\varphi_{i}}{n},\frac{\sum_{i=1}^{n}\lambda_{i}}{n}\right) \end{array}$$

with its standard deviation s

(2) $$\begin{array}{c} s=\left(\sqrt{\frac{1}{n}\sum_{i=1}^{n}{\left(\varphi_{i}-\bar{\varphi_{i}}\right)}^{2}},\sqrt{\frac{1}{n}\sum_{i=1}^{n}{\left(\lambda_{i}-\bar{\lambda_{i}}\right)}^{2}}\right), \end{array}$$

leading to the radius *r*, calculated with the Haversine distance, for indicating the spreading around the center point

(3) $$\begin{array}{c} r=d_{\mathrm{haversine}}\left(C,C+s\right). \end{array}$$

This enables the identification of the general mean location of the anomalies and their distribution density.

## 3. Results and Discussion

### 3.1. Missing Value Imputation

**Figure 3:**
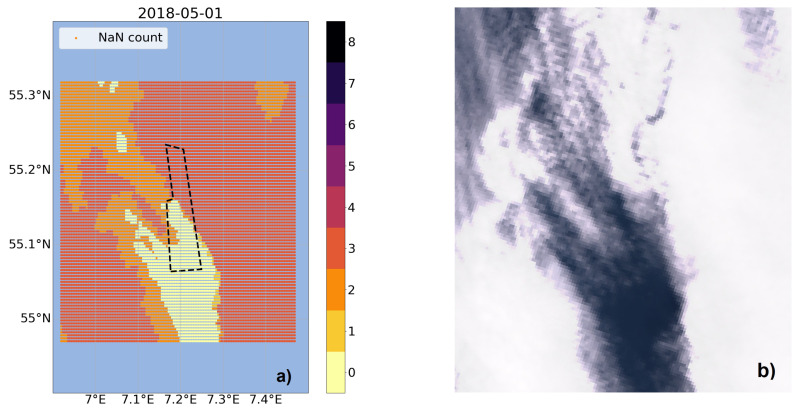
Comparison of missing data: (**a**) Missing value plot of the study area with the color scale indicating the number of features missing for the individual grid point for 1 May 2018. The DanTysk wind farm is marked with a black dashed line. (**b**) Sentinel-3A OLCI image of the DanTysk wind farm area for the same day. Credit: European Union, contains modified Copernicus Sentinel data (2023) processed with EO Browser.

For the gap imputation, the DTWkNN algorithm is implemented and used as an ensemble method for filling gaps in the individual time series of the grid points. First, these gaps need to be analyzed in order to get a better understanding. Therefore, plots showing the missing data for each day individually are created; see Figure 3a as an example.

For each grid point it is analyzed how many features are missing for the day at hand. The results vary with most days having a complete dataset without any values missing or with only single grid points with just one feature missing. For nine of the 31 days there are grid points with up to three parameters missing. These grid points are often adjacent and form patterns. When comparing it with Sentinel-3 OLCI images (e.g., Figure 3b), these structures can be connected to occurring clouds, which are an issue for the satellite measurement and therefore cause unavailability of data. The single grid points with missing data could be filled with a spatial interpolation method just using the information of the neighboring points. As there are areas with multiple grid points containing missing values, a temporal approach is preferred since the neighboring points cannot be used without the necessary information available. When examining the time series for every grid point, there are only individual data points missing referring to one day, which would also be possible to fill with a simple interpolation approach, e.g., linear interpolation.

**Figure 4:**
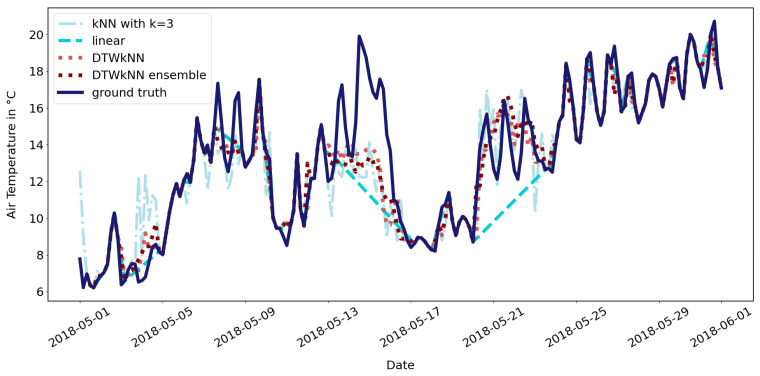
Example of using the DTWkNN as a single and as an ensemble method for gap imputation compared to linear interpolation and kNN. Gaps are artificially induced in every feature of the FINO3 data; the filled results are shown for the air temperature.

The main advantage of the DTWkNN algorithm is the performance regarding the problem of consecutively missing values, which in environmental applications can often be the case due to sensor failure and remote access. With enough data available, the information about the temporal behavior including the temporal variability can be gained, since specific time windows around the gap are compared to all equally sized time windows in the remaining time series. Preliminary results of this method are shown in Figure 4 as an example with artificially induced gaps for the FINO3 meteorological data. Smaller gaps can also be filled with linear interpolation, but larger gaps perform significantly better with more complex algorithm as a kNN with k=3 and the DTWkNN, which catch the temporal variability of the data. In comparison to the DTWkNN, the kNN tends to overfit. $R^{2}$ is 0.64 for both DTWkNN methods, 0.47 for kNN and 0.31 for linear interpolation. With this evaluation of the algorithm on a small dataset, the practicability of the DTWkNN can be shown, which suffices for the purpose of this work. For a more thorough analysis of its performance, a larger dataset would be needed.

**Figure 5:**
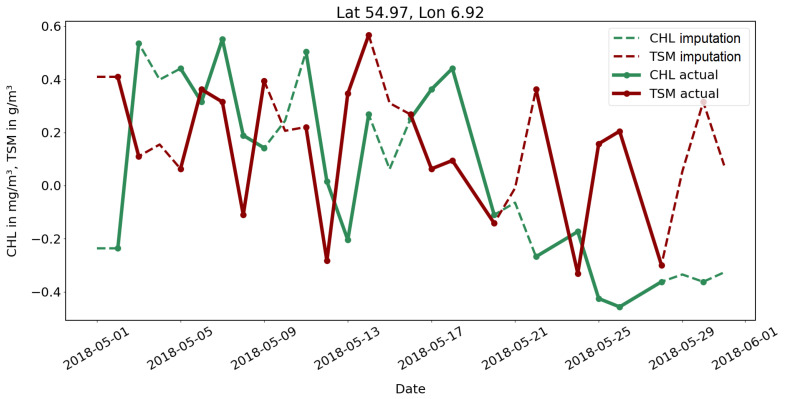
DTWkNN gap imputation exemplary for TSM (red) and CHL (green) time series for one grid point. The continuous lines with the markers show the actual data, whereas the dashed lines show the imputation results.

Finally, this method is used on the original dataset with the actual gaps. The result is demonstrated in Figure 5. Every missing value within the dataset is filled, despite its source. Given that most of the gaps occur in the satellite features, the example shows the imputation for CHL and TSM. Without ground truth data, validation of these results is not possible, but values are accepted for further utilization due to the previous results and the objective of generating a first feasable use case.

### 3.2. Anomaly Detection

**Figure 6:**
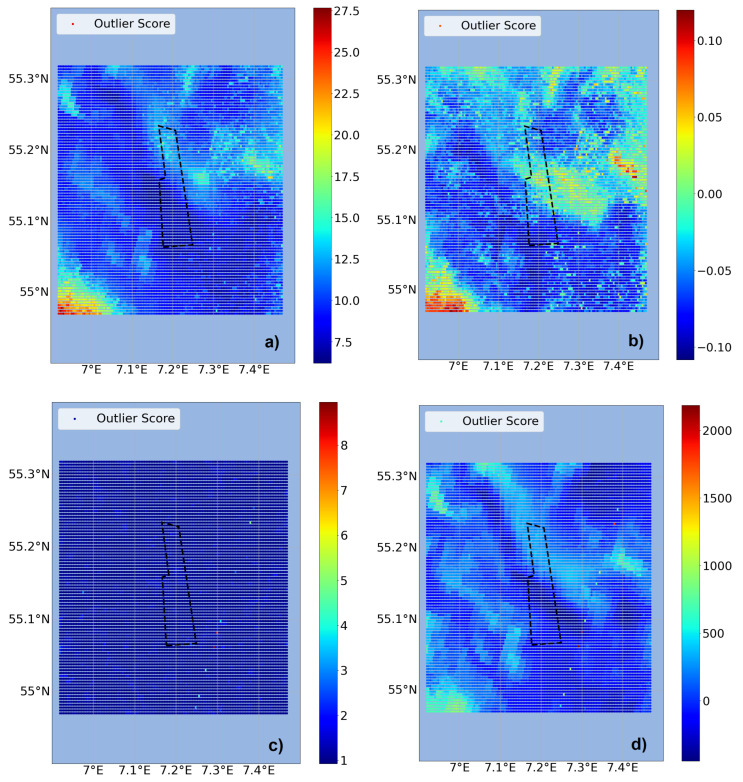
Spatial anomaly detection, exemplary for 8 May 2018. The detectors (**a**) COPOD, (**b**) IForest, (**c**) LOF and (**d**) OCSVM are compared regarding their anomaly score distribution. The color scale values are set by the individual detector. The DanTysk wind farm is marked with a black dashed line.

With the completed dataset the anomaly detection can be performed. As described in the previous section, four anomaly detectors were tested within the two approaches. First, the spatial approach was tested as shown in Figure 6. For this exemplary day COPOD and IForest show structures in the form of clusters in the data but the highest anomalies are located at the edge of the area of interest. LOF only has single anomalous grid points, whereas OCSVM forms clusters but without high values. Basically, this approach connects the already available information from the basic visualization of the single features with possible anomalies but more information content is needed for thorough evaluation. Within this approach it does not make a difference to include or exclude the FINO3 wind data, since it is only a point measurement, so it is valid either to use just one grid point or to give every grid point the same value as it contains the information of the areal weather conditions present.

**Figure 7:**
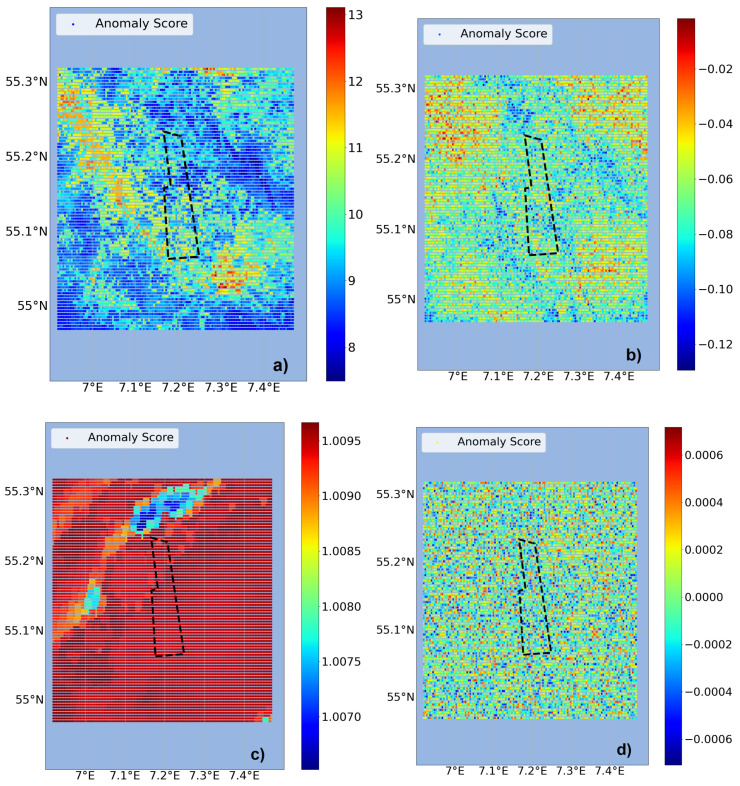
Temporal anomaly detection including FINO3 wind data as input, exemplary for 8 May 2018. The detectors (**a**) COPOD, (**b**) IForest, (**c**) LOF and (**d**) OCSVM are compared regarding their anomaly score distribution. The color scale values are set by the individual detector. The DanTysk wind farm is marked with a black dashed line.

**Figure 8:**
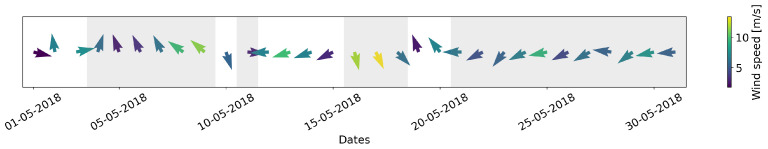
Time series of FINO3 wind data for May 2018. Arrows indicate the wind direction (up = north), whereas the color scale shows the wind speed. Time windows with similar wind conditions are marked as alternating grey/white areas for later analysis.

**Figure 9:**
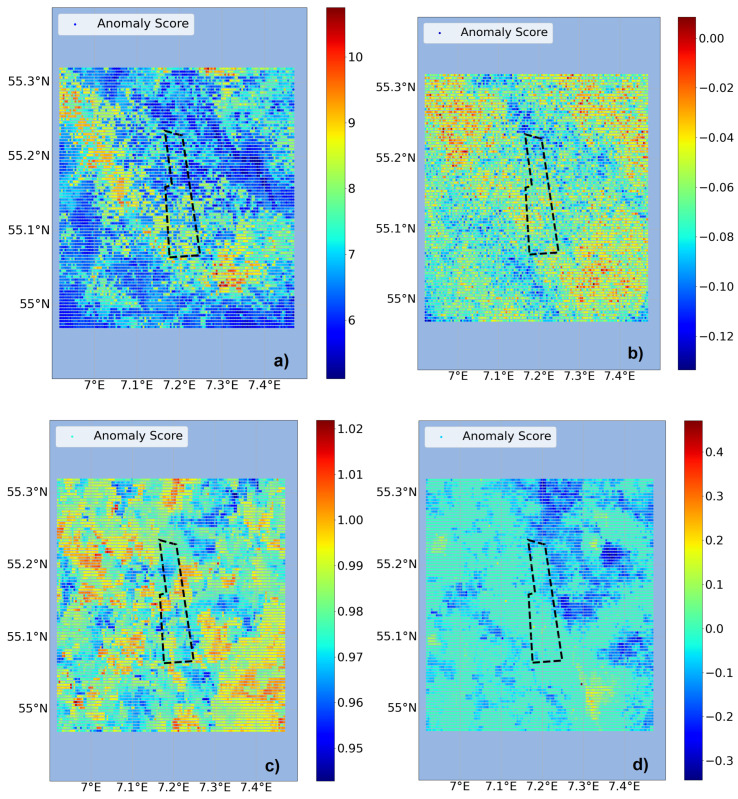
Temporal anomaly detection without FINO3 wind data, exemplary for 8 May 2018. The detectors (**a**) COPOD, (**b**) IForest, (**c**) LOF and (**d**) OCSVM are compared regarding their anomaly score distribution. The color scale values are set by the individual detector. The DanTysk wind farm is marked with a black dashed line.

The second approach is to use temporal anomaly detection and first include the FINO3 wind data as additional input, see Figure 7. COPOD and IForest again show structures and clusters of higher anomalies, mostly arranged along the northwest to southeast line. This matches the wind present on that day (8 May 2018) in the northwest direction, see Figure 8. One of the main drivers of potential effects being when wind forces the surface water in the direction of the wind farm, anomalies in wind direction are relevant for evaluations. LOF shows high anomalies for most of the grid points, whereas for OCSVM only noisy behavior is identifiable. With values fluctuating marginally around zero, OCSVM results in no significant anomalies for this approach. To include the wind information can bias the outcome of the anomaly detectors towards the high parameter values within the wind time series (Figure 8). For example, in mid-May strong winds with a southward and consequently unusual wind direction were present. Such impacts can be reduced with the use of more data as a basis for the time series evaluation. Thus, the approach using temporal anomaly detection but excluding FINO3 data as input is tested, see Figure 9. For all four detectors there are more matching clusters and structural behavior visible. COPOD and IForest still show anomaly structures within the wind direction. In particular, COPOD shows a similar outcome to the previous approach. Therefore, it can be considered the most stable detector in the sense of reproducibility. In addition, greater usability is given for COPOD by the lack of need for hyperparameter tuning compared to the other detectors.

In general, the temporal approach can give additional insight compared to just using the already visual data from the spatial approach, as the data is analyzed with the temporal component. Due to these reasons, the temporal approach without FINO3 data as input and the COPOD algorithm are chosen for further analysis. Still, the wind measurements from FINO3 (Figure 8) are taken as additional information for the interpretation of the anomaly results.

**Figure 10:**
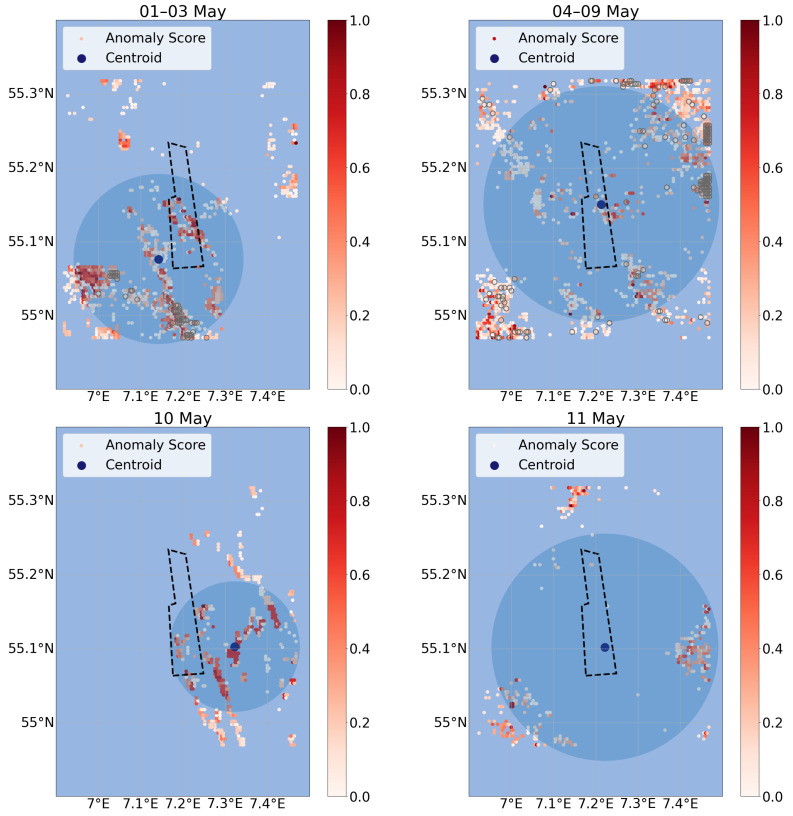

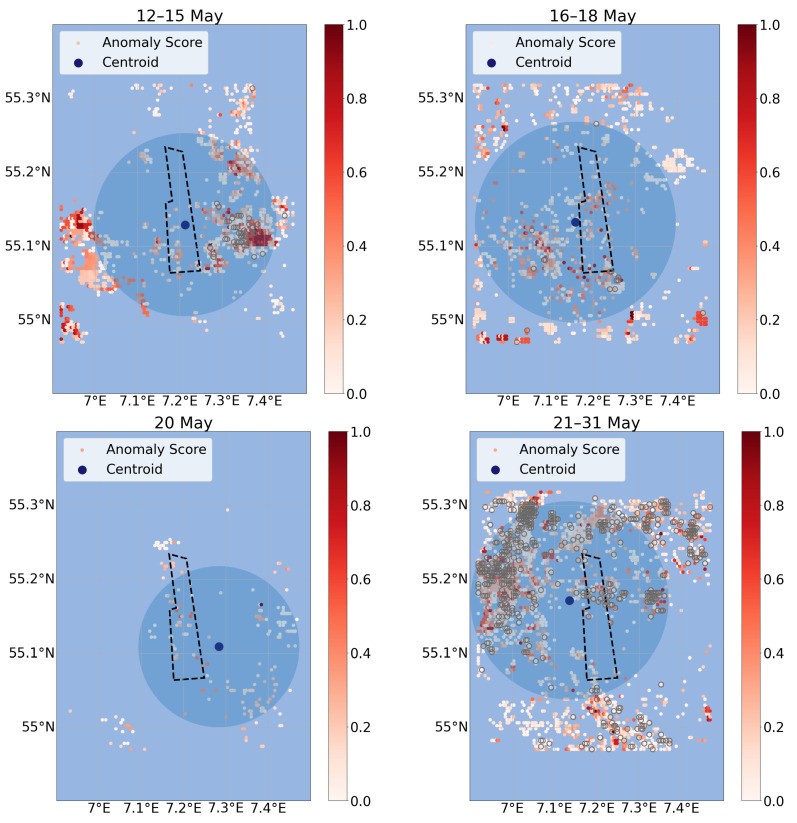
Anomaly maps over specific time windows with similar weather conditions. For each time window, the highest scaled 3% of anomaly scores per day are normalized between 0 and 1 and finally averaged. The DanTysk wind farm is marked with a black dashed line.

To monitor the surroundings of an OWF regarding the effects on the marine environment, the anomalies found in the available data need to be analyzed with respect to position, temporal variability and spatial extent. Since the anomaly detector returns an anomaly score with the probability of a data point being an anomaly, a threshold needs to be set to identify the anomalies most accountable for the effects. In the literature [7,30], effects on the ocean dynamics such as eddies or upwelling/downwelling cells are found to be of similar size as the OWF and last for several days under certain conditions. In accordance with this, the threshold was set by the size of DanTysk which in size equivalents to the grid includes 369 grid points. This corresponds to about 3% of the total amount of 12,144 grid points. Therefore, the highest 3% of the anomaly scores are above the threshold and are counted as impact anomalies. Finally, to match the time span of several days, time windows with similar weather conditions within the month were selected by sorting after similar wind directions (alternating grey/white areas in Figure 8). Over these time windows, the anomaly data were averaged. For better visualization, these values were normalized between 0 and 1. The upper 3% of each day adapted by a scaling factor to minimize bias was taken. As scaling factor the standard deviation is used in order to reflect that a day with a high standard deviation should be considered more anomalous than a day with steady values. The results of these final adjustments can be seen in Figure 10. To get more insight into the anomalous clusters in the remaining data and make these results more quantifiable, a centroid for each time window was calculated together with the standard deviation of locations as a measure for cluster density. With the position of the centroid, the location of the anomalies and consequently the effects can be determined. In addition, grid points which were classified as anomalous multiple times are marked with a grey circle around the data point to underline their impact.

With regards to the wind direction, short-term anomalies can be found in front and behind the wind farm (e.g., Figure 10, 12–15 May), whereas long-term anomalies are more stable behind the wind farm (e.g., Figure 10, 21–31 May). The temporal span of the anomalies is defined by the size of the time windows. Time windows of just one day occur as well, as the weather conditions can change quickly, e.g., Figure 10, 10 May. Here, the highest anomalies can be connected to the edge of the cloud coverage. This effect can be reduced in longer time windows with more data to average over. The long-term anomaly in the data available is given by the last ten days with stable weather conditions. Here, there are in general more anomalies since the mean is calculated over a larger time span compared to the other time windows. Within this time window, the wind direction was on average westward. The centroid of the anomalies can be found on the west side of the wind farm with many anomalies in west, north and south directions, but almost none on the east side. This matches with expectations of effects being behind a wind farm in terms of currents and wind directions. After passing through the wind farm, turbulences in the water can occur which can consequently change the stratification of ocean layers. In general, clusters with a lower standard deviation are denser which makes the location of the anomaly more precise and therefore the results more reliable.

### 3.3. Explainability

For stakeholders, users, application and decision making, digital twins need to be transparent and explainable [13,31,32]. Addressing this problem, a deeper understanding of the origin of the anomalies is important. This can be achieved by using the algorithm’s functionalities. The COPOD detector has the possibility of sorting the parameters’ amount of influence on the resulting anomaly/outlier score, providing a so-called outlier explanation function.

**Figure 11:**
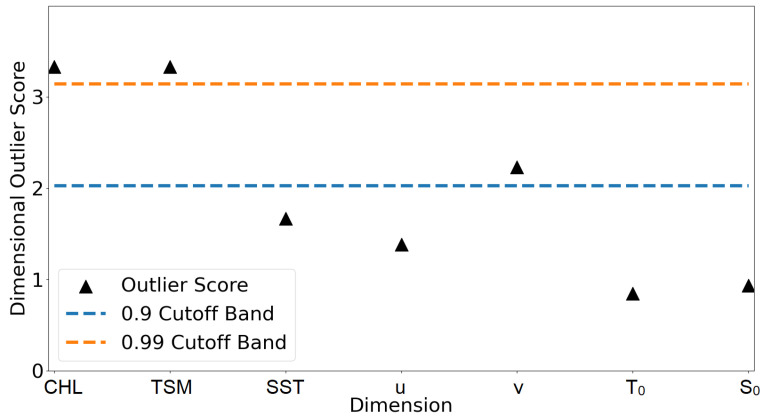
Outlier explanation for one exemplary grid point indicating the feature importance for the calculation of the anomaly score. The highest anomaly within the time series was evaluated (sample 18). The features CHL, TSM and SST are Sentinel products, whereas *u*, *v*, $T_{0}$ and $S_{0}$ originate from the hydrodynamical model.

**Figure 12:**
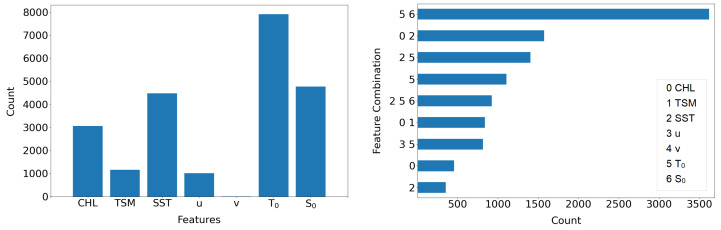
Statistics on outlier explanation. Responsibility of features (**left**) and their most frequent combinations (**right**) for the highest anomaly in each time series of a grid point. The features CHL, TSM and SST are Sentinel products, whereas *u*, *v*, $T_{0}$ and $S_{0}$ originate from the hydrodynamical model.

For each grid point, the highest anomaly score in the time series was identified and evaluated with this function, see Figure 11. Sample number 18 (referring to 18 May 2018), was found to be the day with the highest anomaly in the time series of this grid point. This sample’s anomaly score can be attributed to the parameters CHL and TSM, as displayed as values above the 0.99 cutoff band. This method is evaluated for every grid point and statistically evaluated, see Figure 12.

The results give insight only on the highest anomalies in the time series of each grid point, showing the combination of features as well as the individual feature results. Since the data are interconnected, this value is often referring to the same day. Hence, some of the features occur more often than others, e.g., the model feature water temperature $T_{0}$. An explanation for this, besides it being a real anomaly, can be the hydrodynamical model’s uncertainty. The model does not yet include the information of wind farms in its calculations. If there is an effect due to the OWF, the satellite data might deviate from the model data more than on days without major effects. The anomaly detection can then be influenced by this discrepancy. Anomalies that can be traced back to the satellite data are more valuable for this demonstrator, as they contain the wind farm information. In general, the temperature seems to be the key factor on both sides, satellite and model data. This can be connected to the effect of turbulences and extended mixing, which can have a large influence on the surface temperature.

## 4. Conclusions

**Figure 13:**
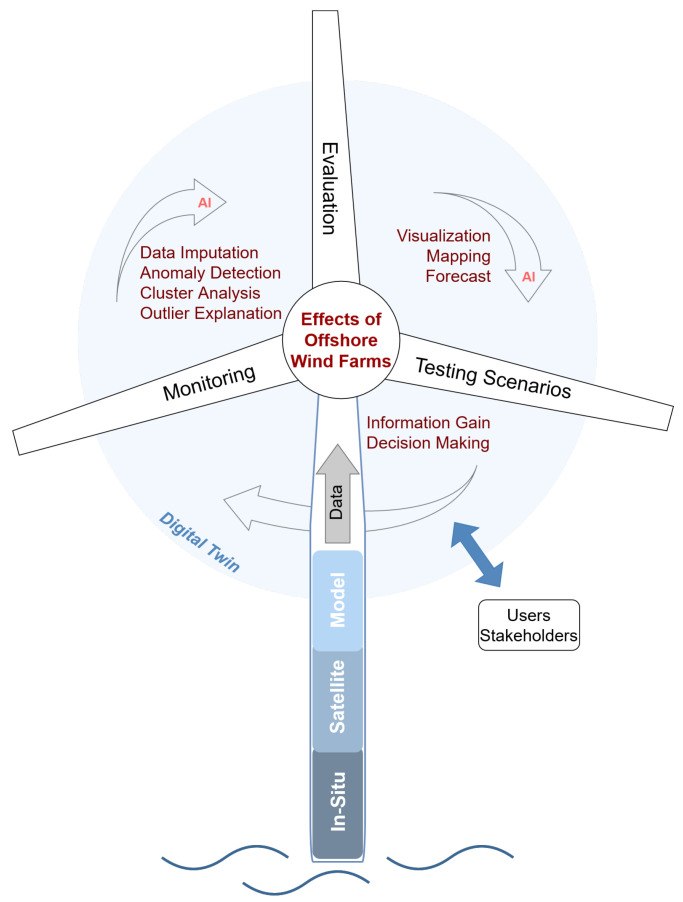
Effects of OWFs on the marine environment in the context of a digital twin. Data from different sources form the basis for monitoring, evaluation and testing scenarios. Steps in between can be performed with AI algorithms, leading to information gain and decisions, based upon which the cycle can start over. Users and stakeholders can interact with this system for applications and decision making.

Monitoring changes in the ocean currents and in the stratification of ocean layers due to OWFs, including all the associated processes, represents one of the major challenges for the expansion of offshore green energy. Future considerations and decisions will need to be based upon such results. Therefore, a method is needed, which should be accessible and transparent for users and stakeholders. This suits the idea of a digital twin for environmental applications, where questions can be answered and future scenarios can be run through [15,33]. With interventions for example in location assessment or sizing based upon previous monitoring results from the digital twin, effects of OWFs on the marine environment could be regulated in the real twin (Figure 13).

Here, a tailored application workflow based on easily available data in addition to an automatable process with existing tools is presented. Spatial and temporal behavior of anomalies around the OWF put into context with the wind directions at hand are a valuable outcome for determination of potential effects of the OWF. For longer time windows with stable weather conditions as shown here for 21–31 May 2018, the mean location of anomalies and their distribution northwest of DanTysk fits to the mean wind direction being westward, leading to effects leeside of the wind farm. With proper validation and development, it could advance a continuous monitoring process for OWFs and their marine surroundings. The data for this feasibility study covered only the short amount of time of one month. The ocean as marine ecosystem is turbulent with many processes and variability. Within the North Sea for example, there is a stable stratification of ocean layers in summers [10]. This changes throughout the year and depends on different factors and parameters. Therefore, data need to cover this interdependency on various temporal and spatial scales by using longer time series of at least one year. The challenge then is to delimit the anomaly results from the natural variability [7]. To date, no baseline for the identification of OWF effects is established. Dedicated measurement campaigns for collecting in situ data as ground truth, domain knowledge and with that validation of such effects could further improve the results and their interpretation and should be explored in future work. Including historical data for an OWF area and comparing the stages before the construction of the OWF and afterwards could provide additional information. One challenge regarding the satellite data is dealing with clouds, which could be reduced by combining different satellites and their products [34,35]. In general, satellite data come with many challenges but are a strong tool towards the development of digital twins due to their continuous availability and coverage.

Machine learning, as shown here, is a powerful instrument for tasks that deal with data in any forms [15,36], from Big Data to fragmentary data, which both exist in environmental science. Data fusion, preprocessing, examination and predictions are the essential fields where machine learning can contribute the most in digital twins for the environment. The DTWkNN algorithm showed great potential for large gaps in multivariate time series data. In order to meet requirements due to differences in gap origins and characteristics, a combination of multiple gap filling approaches could be a solution. This would also support the generalizability of such monitoring techniques. Being usable and customizable not only for OWF-related application scenarios but also for various environmental applications, especially in the digital twin context, this tackles the challenge of the need for standardization [37]. For anomalies in the data, a temporal evaluation provides additional information, as demonstrated here and in other studies, e.g., hurricane tracking [38] or levee health monitoring [39]. COPOD is found to be a suitable anomaly detector for this application. Being a “parameter-free, highly interpretable and computationally efficient algorithm” [26], COPOD scored here especially due to usability in the form of an explanation function and reproducibility of results despite varying data input. In future works, these fields can benefit by using more optimized tools, e.g., the approach of evaluating the anomalies can be further developed by using AI based cluster analysis and thus automate the evaluation process (Figure 13). With this, multiple center points could be set in order to separate the data into clusters and classify them matching different occurrences. The outlier explanation tool is a strong feature for transparent and explainable insight, and should be included and improved in further work. Finally, predictions combined with visualizations are naturally the main component of testing scenarios.

Within this feasibility study, a workflow for monitoring effects in and around OWFs using machine learning was developed. Different data sources and algorithms for this application were explored and successfully implemented on a functional basis. With the new evaluation technique, the temporal and spatial behavior of detected anomalies could be identified. Analyzing the effects of OWFs on their environment is itself not easily possible without detailed domain knowledge and dedicated measurements representing multiyear variability. However, the discussed machine learning tools and the anomaly detection specifically show potential and can be used to find and visualize important information within the multivariate dataset. The interpretation of such findings will only be possible with explainable methods, as demonstrated here.

For the planned further expansions of offshore renewable energy, reliable monitoring of OWFs and their effects on the environment needs to be established to provide transparent information on the current state but also on possible future scenarios. This is necessary to enable informed decisions, shaping our future of offshore energy infrastructure. By focusing on explainability in the methods generating said information, digital twins can be a valuable tool for these decision making processes.

## Figures and Tables

**Table 1:** Overview of data types and parameter properties used for the study.

	Sentinel-3	BSH-HBMnoku	FINO3
Type	Satellite	Model	In situ
Variable	Sea surface temperature Total suspended matter Chlorophyll	Temperature Salinity Current velocity Current direction	Wind speed Wind direction
Spatial resolution	300 m–1 km	900 m	Point measurement
Temporal resolution	1 d	15 min–1 h	10 min

## Data Availability

Sentinel data products are available through Copernicus Open Access Hub (https://scihub.copernicus.eu/, accessed on 1 March 2023). In situ data from FINO are published in the FINO database (http://fino.bsh.de, accessed on 1 March 2023). Data from the hydrodynamical model are provided by BSH (https://www.bsh.de/DE/THEMEN/Modelle/modelle_node.html, accessed on 1 March 2023).

## Abbreviations

The following abbreviations are used in this manuscript:

AI	Artificial Intelligence
CHL	Chlorophyll
COPOD	Copula-Based Outlier Detection
DTWkNN	Dynamic Time Warping k-Nearest Neighbor
IForest	Isolation Forest
LOF	Local Outlier Factor
OCSVM	One-Class Support Vector Machine
OLCI	Ocean and Land Color Instrument
OWF	Offshore Wind Farm
SLSTR	Sea and Land Surface Temperature Radiometer
SSS	Sea Surface Salinity
SST	Sea Surface Temperature
TSM	Total Suspended Matter
